# Isoniazid potentiates tigecycline to kill methicillin-resistant *Staphylococcus aureus*

**DOI:** 10.1080/22221751.2024.2434587

**Published:** 2024-11-25

**Authors:** Xuan-wei Chen, Hao-qing Chen, Jia-han Wu, Zhi-han Wang, Yu-Qing Zhou, Si-qi Tian, Bo Peng

**Affiliations:** a State Key Laboratory of Bio-Control, Guangdong Key Laboratory of Pharmaceutical Functional Genes, School of Life Sciences, Southern Marine Science and Engineering Guangdong Laboratory (Zhuhai), Sun Yat-sen University, Guangzhou, People’s Republic of China; b Laboratory for Marine Biology and Biotechnology, Qingdao Marine Science and Technology Center, Qingdao, People’s Republic of China; c Guangzhou Medical University, Guangzhou, People’s Republic of China

**Keywords:** Tigecycline, isoniazid, methicillin-resistant *Staphylococcus aureus*, proton motive force, antibiotic influx

## Abstract

Therapeutic option for treating methicillin-resistant *Staphylococcus aureus* (MRSA) infection is urgently required since its resistance to a broad spectrum of currently available antibiotics. Here, we report that isoniazid is able to potentiate the killing efficacy of tigecycline to MRSA. The combination of isoniazid and tigecycline reduces the minimal inhibitory concentration of clinic MRSA strains to tigecycline. The killing activity of tigecycline is further confirmed by killing experiments and murine infection model. We further demonstrate the mechanism that isoniazid increases intracellular accumulation of tigecycline by promoting the influx but limiting the efflux of tigecycline through proton motive force. We also show that isoniazid and tigecycline synergize to increase the abundance of isoniazid-NAD adduct, which in turn damage cell membrane, possibly contributing to the disruption of PMF. Whereas phosphatidylethanolamine and cardiolipin are able to abrogate the synergistic effect of isoniazid plus tigecycline. Thus our study provides a new perspective that antibiotics, e.g. isoniazid, once recognized only to target *Mycobacterium tuberculosis*, can be repurposed as antibiotic adjuvant to tigecycline, expanding our choice of antibiotic–antibiotic combinations in treating bacterial infectious diseases.

## Introduction

Methicillin-resistant *Staphylococcus aureus* (MRSA) is one of the notorious bacterial pathogens that are difficult-to-treat in clinic [1]. MRSA is the primary pathogen leading to skin and soft tissue infections, bacteraemia and joint infection, which are common in community- and hospital-acquired infection [2]. World Health Organization announced MRSA is one of the six multidrug-resistant pathogens required for urgent attention [3]. The incidence of MRSA infection is 20–30 cases for every 100,000 population in high-income countries each year, and the number is believed to be increasing in recent years [4,5]. Clinic investigations show that the mortality rate of patients suffered from MRSA infection and infective endocarditis is 17–50% [6]. Meanwhile, MRSA is also a threat to agriculture. The infection of economic animals including pigs, cattle, poultry, and even in aquatic products, poses big economic burden to sustainable agriculture [7–9]. More importantly, MRSA with higher virulence can be transmitted between animals and human beings, threatening human health via food chain [10]. Thus expanding therapeutic options to treat MRSA infection is urgently required.

The treatment of MRSA is challenging because of its resistance to a broad spectrum of antibiotics. Vancomycin or teicoplanin, the last line of defence against MRSA, leads to the emergence of corresponding resistant MRSA [11,12]. Other antibiotics like tigecycline (TIG), linezolid and daptomycin are also used to treat MRSA, but they have limited therapeutic effects [13]. Meanwhile, TIG should be used in caution due to its resistance was mainly mediated through efflux pump [14]. Several studies have indicated the use of TIG, for example, had high frequency of adverse events and is associated with high mortality [15,16]. Thus development of method that improves the killing efficacy and decreases side effect is necessary to manage MRSA infection.

Isoniazid (INA) is a synthetic antibacterial drug with bactericidal effect to *Mycobacterium tuberculosis* (MTB) [17]. INA has high specificity to inhibit the growth of MTB through disrupting the synthesis of mycobacteric acid, leading to cell wall rupture [18], but had limited effect to other bacteria [19]. It has also been shown that INA can be combined with other antibiotics including amikacin, cefotaxime, or imipenem to treat MTB and reduce the occurrence of resistance [20]. However, the use of INA alone or use with other antibiotics is limited to MTB, whereas research is rarely conducted on whether INA–antibiotic combination can be used to treat other types of infection. Here, we find that INA can be used as a TIG adjuvant that potentiates TIG killing efficacy of MRSA, proposing another potential use of INA in clinic.

## Materials and methods

### Bacterial strains and growth conditions

All clinic MRSA strains were isolated from The Third Affiliated Hospital of Sun Yat-sen University. MRSA strain, ATCC29213, was purchased from American Tissue Culture Center. Strains of *E. coli*, *P. aeruginosa*, *K. pneumonia* and *S. agalactiae* are our lab stocks [21,22]. To propagate bacterial growth, a single colony was picked up and inoculated into Luria–Bertani (LB) medium or brain heart infusion (BHI) medium with shaking.

### Fractional inhibitory concentration index (FICI) determination

FICI was determined by checkerboard assays as previously described [23,24]. Briefly, INA and TIG were serially diluted. Then 1 × 10^5^ CFU/mL bacterial suspensions were added into each well. After culturing at 37°C for 16 h, the MICs were recorded as the lowest concentration of drug inhibiting visible growth. The synergistic effect was determined by calculating FIC according to the formula: FICI = MIC_AB_/MIC_A_ + MIC_BA_/MIC_B_ = FIC_A_ + FIC_B_. MIC_A_ is the MIC of compound A alone; MIC_AB_ is the MIC of compound A in combination with compound B; MIC_B_ is the MIC of compound B alone; MIC_BA_ is the MIC of compound B in combination with compound A; FIC_A_ is the FIC of compound A; FIC_B_ is the FIC of compound B. The synergy or additive was defined according to standard criteria (FICI ≤ 0.5 was defined as synergistic; 0.5 < FICI ≤ 1 was defined as additive; 1 < FICI ≤ 4 was defined as indifference; FICI > 4 was defined as antagonism).

### Antibiotic killing assay

Antibiotic killing assay was performed as previously described [25]. Briefly, bacterial cells were collected by centrifugation at 8000 rpm for 3 min. The samples were then washed with sterile saline three times and resuspended in LB medium, diluted to OD_600_ of 0.2 and subjected to a 100-fold dilution, then incubated with INA or/and TIG at 37°C for 6 h with shaking. To determine bacterial counts, 100 μL of cultures was removed from each treatment and then serially diluted at 10 times in sterile saline buffer. An aliquot of 5 μL of each dilution was spotted on LB agar plates and incubated at 37°C for 16 h. The dilution containing 20–80 colonies would be used to determine the CFU. The percent of survival was calculated based on the CFU of experimental group to control group.

### Generation of TIG-resistant MRSA

The generation of antibiotic-resistant MRSA was performed as previously described [26]. Briefly, a single colony of MRSA7 or MRSA11 was selected and cultured in LB medium at 37°C with shaking. Overnight cultures were transferred into fresh growth medium at 1:100 plus 1/2 MIC TIG of the corresponding strains, e.g. 0.125 μg/mL for MRSA7 and 0.25 μg/mL for MRSA11, which was cultured at 37°C with shaking. The bacteria was passaged every 24 h, and MIC was monitored every three passages. The concentration of TIG in culture medium was adjusted based on the change of MIC. MRSA7-R_TIG_ and MRSA11-R_TIG_ were obtained when MIC was increased for 32 folds.

### Preparation of persisters

The preparation of persisters was performed as previously described [27,28]. Overnight cultures of MRSA were collected by centrifugation at 8000 rpm for 3 min. The samples were then washed with sterile saline three times and resuspended in LB medium, then incubated with 5 μg/mL ciprofloxacin for 6 h. Then, bacteria was treated with higher dose of ciprofloxacin for 4 h. Bacterial viability was checked every hour to ensure there is no more bacterial death. Subsequently, persisters were proceeded for antibiotic killing assay.

### Preparation of biofilm

Biofilm-formation culture was determined as previously described [27]. Briefly, 6 mm PE50 catheters (0.5 mm × 1 mm) were inoculated in 1 mL fresh LB and 10 μL cultured stationary phase bacteria, then incubated aerobically for 24 h at 37°C. The medium was changed every 24 h for a total of 3 days. The PE50 catheters were washed three times with 1 mL of sterile saline to remove loosely adherent cells and planktonic cells. Then the catheters were proceeded for antibiotic killing assay.

### Mice infection experiment

To evaluate the synergistic effect of INA and TIG *in vivo*, Kunming mice with high genetic heterozygosity was used as previously adopted [29,30]. Kunming mice were obtained from the Laboratory Animal Center of Sun Yat-sen University (Guangzhou, China), with an average age of 4 weeks and an average weight of 20 g. A total of 40 mice were randomly divided into four groups with 10 mice at each group. Each mice was injected 1 × 10^8^ CFU of MRSA via intraperitoneal injection followed by injection with a single dose of TIG at 10 mg/kg, INA at 100 mg/kg, or TIG combined with INA. The last group was treated saline buffer as control. At 12 h, mouse liver, kidney, and spleen were separately collected and homogenized. Supernatant was used to examine bacterial cells via viable plate counting.

### Determination of minimum inhibitory concentration (MIC) and minimum bactericidal concentration (MBC)

The determination of MIC was performed as previously described [31]. To determine MIC, an overnight bacterial culture was diluted 1:100 (v/v) in fresh broth medium and cultured at 37°C to an OD_600_ of 0.2. Then cells equivalent to 10^5^ CFU were dispensed into each well of a 96-well microtiter polystyrene tray after which a series of twofold dilutions of antibiotic was added. Following incubation at 37°C for 16 h, the MIC was defined as the lowest antibiotic concentration that inhibited visible growth. Three biological repeats were carried out.

The determination of MBC was performed as previously described [32]. MBC is the lowest concentration that kills 99.9% of bacteria after 24 h incubation at 37°C. MBC values were determined by removing 3.5 μL of bacterial suspension from the well of MIC plate that has no visible growth and plating in the agar plate to determine CFU. Plates were incubated at 37°C for a total period of 16 h. Each experiment was repeated at least three times.

### Quantification of intracellular TIG

The quantification of intracellular antibiotic content was performed as previously described [33]. Briefly, bacteria were incubated with TIG or INA or both at 37°C for 6 h and then collected by centrifugation at 8000 rpm for 3 min. Bacterial pellets were washed three times with sterile saline buffer and resuspended in the same buffer to OD_600_ = 1.0. A total of 25 mL samples were sonicated for 10 min, centrifuged and the supernatant was carefully removed to a new tube. Then the sample was diluted, added to the 96-well immunoplates (Cat. 95371O1, CSM, China) coated with 50 μL antibody, and incubated at 37°C for 30 min. The plates were washed five times and then reacted with 50 μL of substrate A solution and B solution at 37°C for 10 min in dark. The reaction was stopped by stopping solution. The absorbance at 450 nm was measured in a PerkinElmer LS55 Fluorescence Spectrophotometer (PerkinElmer). The quantity of TIG was determined by standard curve. Three biological replicates were included for treatment.

### Quantitative real-time polymerase chain reaction (qRT-PCR)

qRT-PCR was carried out as previously described [31]. 1 mL of bacterial culture with OD600 = 1.0 was harvested. The Trizol (Invitrogen, United States) was used to isolated total RNA of each sample. Then, an EvoM-MLV RT kit with gDNA clean for qPCR (AG11705; Accurate Biotechnology) was used to reverse transcription-PCR. qRT-PCR was performed in 384-well plates with a total volume of 10 μL containing 5 μL 2× SYBR green premix pro Taq HS qPCR kit (AG11701; Accurate Biotechnology), 2.6 μL H2O, 2 μL cDNA template, and 0.2 μL each of forward and reverse primers (10 mM). The primers are listed in Supplementary Table 1. All samples were tested in biological triplicate and run on the LightCycler 480 system (Roche, Germany) according to the manufacturer’s instructions, and four independent samples were assayed for both the control group and the test group. The cycling parameters were 95°C for 30 s to activate the polymerase; 40 cycles of 95°C for 10 s; and 56°C for 30 s. Fluorescence measurements were performed at 72°C for 1 s during each cycle. Cycling was terminated at 95°C with a calefactive velocity of 5°C/s to obtain a melting curve. Data are shown as the relative mRNA expression compared with control with the endogenous reference 16S rRNA gene. Primers used in this study are listed in Suppl. Table 1.

### Propyl iodide (PI) test

PI staining was performed as previously described [34]. PI was determined by a Propidium iodide (PI) fluorescent probe (Cat. P3566; Invitrogen, USA). In brief, *MRSA* cells were resuspended to an OD_600_ of 0.2 and subjected to a 100-fold dilution, then incubated with INA or/and TIG at 37°C for 6 h. After washing with PBS (pH 7.4) three times and resuspending to obtain an OD_600_ of 0.2, 1 mL bacterial solution was absorbed and divided into 1.5 mL EP tubes. 2 μg/mL of PI dye solution was added to each tube, thoroughly mixed and incubated at 37°C for 30 min in the absence of light. The fluorescence value was determined by a flow cytometry with a wavelength of 544 nm and emission light at 620 nm.

### Calcein leakage assay

Calcein leakage assay was performed as previously described [34]. Calcein was determined by a polyanionic fluorescein derivative (Cat. C481; Invitrogen, USA). In brief, MRSA cells were resuspended to an OD600 of 0.2 and subjected to a 100-fold dilution, then incubated with INA or/and TIG at 37°C for 6 h. After washing with PBS (pH 7.4) three times and resuspending to obtain an OD600 of 0.2, 1 mL bacterial solution was absorbed and divided into 1.5 mL EP tubes. 2 μg/mL of Calcein dye solution was added to each tube, thoroughly mixed and incubated at 37°C for 30 min in the absence of light. The fluorescence value was determined by a flow cytometry with a wavelength of 490 nm and emission light at 517 nm.

### N-phenyl-1-naphthylamine (NPN) uptake assay

NPN uptake assay was performed as previously described [35]. NPN fluorescent probe (Cat. 104043; Sigma, USA). In brief, *MRSA* cells were resuspended to an OD_600_ of 0.2 and subjected to a 100-fold dilution, then incubated with INA or/ and TIG at 37°C for 6 h. After washing with PBS (pH 7.4) three times and resuspending to obtain an OD_600_ of 0.2, 1 mL bacterial solution was absorbed and divided into 1.5 mL EP tubes. 10 μg/mL of NPN dye solution was added to each tube, thoroughly mixed and incubated at 37°C for 30 min in the absence of light. The fluorescence value was determined by a fluorescent enzyme labelling instrument with a wavelength of 350 nm and emission light at 420 nm.

### Measurement of Membrane Potential

Membrane potential was measured as previously described [36]. Bacterial membrane potential was determined by a fluorescent probe DiOC2 [3] (Cat. M34150; Invitrogen, USA). In brief, *MRSA* cells were resuspended to an OD_600_ of 0.2 and subjected to a 100-fold dilution, then incubated with INA or/and TIG at 37°C for 6 h. After washing with PBS (pH 7.4) three times and resuspending to obtain an OD_600_ of 0.2, 1 mL bacterial solution was absorbed and divided into 1.5 mL EP tubes. 10 μL of 3 mM DiOC2 [3] dye solution was added to each tube, thoroughly mixed and incubated at 37°C for 30 min in the absence of light. The fluorescence value was determined by an FACSCalibur flow cytometer (Becton Dickinson, San Jose, CA) through a 488−530/610 nm bandwidth band-pass filter, respectively.

### Measurement of pH

pH was measured as previously described [36]. Bacterial intracellular pH was determined by the pH-sensitive fluorescence probe BCECF-AM (Cat. BL929A, Biosharp, China). In brief, *MRSA* cells were resuspended to an OD_600_ of 0.2 and subjected to a 100-fold dilution, then incubated with INA or/and TIG at 37°C for 6 h. After washing with PBS (pH 7.4) three times and resuspending to obtain an OD_600_ of 0.2, 1 mL bacterial solution was absorbed and divided into 1.5 mL EP tubes. 10 μM BCECF-AM dye solution was added to each tube, thoroughly mixed and incubated at 37°C for 30 min in the absence of light. The fluorescence value was determined by an FACSCalibur flow cytometer (Becton Dickinson, San Jose, CA) through a 488−535 nm bandwidth band-pass filter, respectively.

### Measurement of INA-NAD Adduct

The formation of INA-NAD adduct was performed as previously described [37,38]. Briefly, MRSA cells were resuspended to an OD600 of 0.2 and subjected to a 100-fold dilution, then incubated with INA or/ and TIG at 37°C for 6 h. After washing with PBS (pH 7.4) three times and resuspending to obtain an OD600 of 1.0, 20 mL bacterial solution was absorbed and divided into 1.5 mL EP tubes. And the optical density (OD) of INA-NAD was measured by spectrophotometer (Thermo Scientific NanoDrop). In comparison with the absorption spectrum, increases in absorbance at 300–350 nm and the A 326/A 278 ratio were used as indicators for the complex formation.

## Results

### Isoniazid (INA) potentiates tigecycline (TIG) to methicillin-resistant *Staphylococcus aureus* (MRSA)

**Figure 1.**
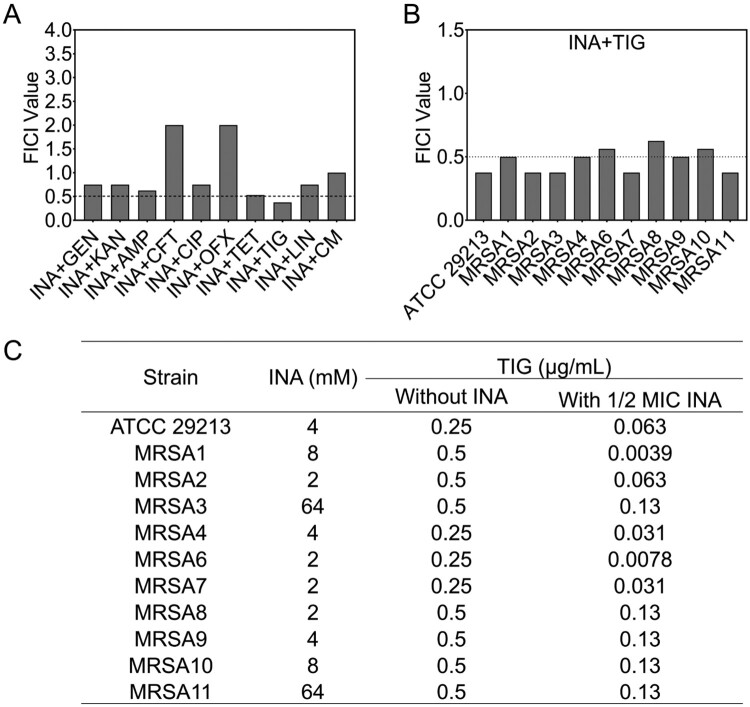
INA potentiates TIG to eliminate MRSA. (A) The FICI of INA combined with different types of antibiotics. (B) The FICI of INA combined with TIG to different MRSA strains. (C) The MIC of MRSA to TIG in the presence of 1/2 MIC of INA. INA, isoniazid; GEN, gentamycin; KAN, kanamycin; AMP, ampicillin; CFT, ceftaxime; CIP, ciprofloxacin; OFX, ofloxacin; TET, tetracycline; TIG, tigecycline; LIN, lincomycin; CM, chloramphenicol.

To investigate whether INA is able to synergize with different types of antibiotics to treat MRSA infection, we used a clinical isolate of multidrug-resistant MRSA strain, MRSA-7, for the initial screening (Suppl. Figure 1). Fractional inhibitory concentration index (FICI) was used to evaluate the possible synergistic effect of INA with aminoglycosides (gentamycin and kanamycin), β-lactam (ampicillin and cefotaxime), quinolones (ciprofloxacin and ofloxacin), tetracyclines (tetracycline), glycylcycline (tigecycline), lincosamides (lincomycin), and chloramphenicols (chloramphenicol). The combination was evaluated, where FICI ≤ 0.5, 0.5 < FICI < 1, FICI = 1, 1 < FICI < 4, and FICI ≥ 4 were considered as synergism, partial synergism, additivity, indifference, and antagonism, respectively [23,24]. INA was synergized with TIG, while was partially synergized with aminoglycosides, tetracycline and lincomycin, and had no effect on other antibiotics (Figure 1A).

To further confirm the synergistic effect of INA and TIG, we treated 1 standard strain of MRSA, ATCC29213 and 10 clinic multidrug-resistant isolates of MRSA with INA plus TIG and (see Suppl. Figure 1). INA and TIG were synergistic for 8 of the 11 strains and were partial synergistic for another 3 strains (Figure 1B). Furthermore, we measured the MIC of the MRSA strains to TIG in the presence of INA. The presence of 1/2 MIC INA decreased the MIC of MRSA to TIG for 4–128 folds (Figure 1C). To investigate whether INA plus TIG had effect on strains with higher tigecycline resistance, the strains of MRSA7 and MRSA11 were propagated to generate tigecycline-resistant strains, MRSA7-R_TIG_ and MRSA11-R_TIG_, whose MIC to TIG was increased to 8 and 16 μg/mL, respectively, being 32 folds of increase (see Suppl. Figure 2A). Being consistent, the presence of INA decreased the MIC for 16 folds for both of the strains (see Suppl. Figure 2A).

Furthermore, we tested the synergistic effects of INA and TIG to other bacterial pathogens including Gram-negative pathogens, *Escherichia coli*, *Klebsiella pneumonia*, and *Pseudomonas aeruginosa*, and Gram-positive pathogens *Bacillus subtilis*, *Enterococcus faecium,* and *Streptococcus agalactiae*. Interestingly, INA and TIG showed good synergistic effect against all of the three *E. coli* strains, 1 strain of *B. subtilis* and 3 out of 6 strains of *E. faecium* but only partially synergistic to other pathogens (see Suppl. Figure 2B). Thus we only focused on MRSA in this study.

### INA and TIG eliminate MRSA infection *in vitro* and *in vivo*

**Figure 2.**
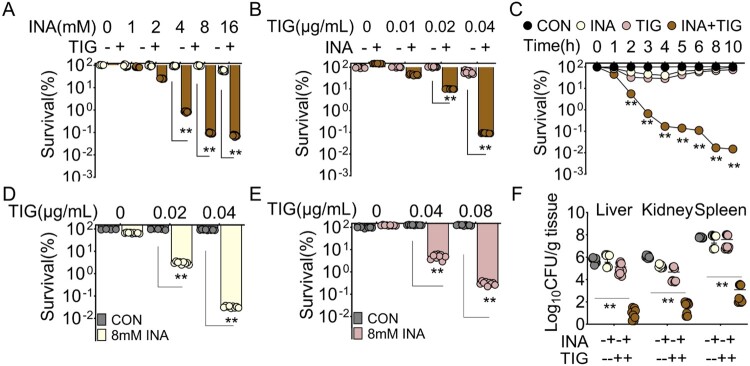
INA and TIG eliminate MRSA infection *in vitro* and *in vivo*. (A) Percent survival of MRSA11 in the presence of the increasing concentration of INA plus 0.04 μg/mL TIG. (B) Percent survival of MRSA11 in the presence of the increasing concentration of TIG plus 8 mM INA. (C) Percent survival of MRSA11 in the presence of 8 mM INA plus 0.04 μg/mL TIG at indicated incubation time points. (D) Percent survival of persister in the presence of INA plus TIG. (E) Percent survival of biofilm in the presence of INA plus TIG. (F) Bacterial loads of mice infected with MRSA in the presence or absence of INA, TIG or both. Results are displayed as mean ± standard errors of the means (SEM) (*N* ≥ 3 technical replicates per sample), and statistically significant differences are identified by Student’s *t* test. *, *p* < 0.05, **, *p* < 0.01. Each experiment was repeated independently at least three times.

To further analyse the synergistic effect of INA and TIG, we treated the strain, MRSA11, with sublethal dose of TIG and INA. When fixed at 0.04 μg/mL TIG, the viability of MRSA was decreased along with the increasing concentration of INA in a dose-dependent manner (Figure 2A), where INA had no killing effect at all tested concentrations. Since INA at 8 and 16 mM had similar potentiation effect, we chose 8 mM INA for the following studies (Figure 2A). Similarly, when the concentration INA was fixed at 8 mM, the viability of MRSA was reduced in a dose-dependent manner (Figure 2B). TIG alone failed to kill MRSA at all tested concentrations. Furthermore, the synergistic effect of INA and TIG was tested in a time-dependent killing assay. If incubated for 8 h or longer, INA increased TIG killing for 3998 folds (Figure 2C). This potentiation suggests that INA may reduce the dosage of TIG that can achieve the same effect. Thus we determined the combination index (CI) and dose-reduction index (DRI) of the INA and TIG combinations. CI indicates the combining effect of drugs, where CI < 1 is considered as synergistic. The CI values for all the combinations of INA and TIG are less than 1, suggestion synergy (see Suppl. Figure 3A). DRI represents the fold of reduced dose when a drug is used in combination with another drug to achieve the same efficacy (Fa) as the drug alone, where DRI > 1 is favourable [25,39]. As such, we only considered Fa > 0.95, where the dosage of TIG was reduced at least nine folds in the presence of INA (see Suppl. Figure 3B). These data suggest that INA may reduce the dosage of TIG to treat MRSA infection.

MRSA infection is always accompanied with persister and biofilm. To investigate whether INA could potentiate TIG on these two situations. MRSA11 was prepared as persister. When TIG was at either 0.02 or 0.04 μg/mL, persister cannot be eliminated. Whereas the supplementation of INA could increase the killing of persister for 3045 folds (Figure 2D). Similar results were observed for the killing of biofilm, where the biofilm cannot be killed by TIG alone even the concentration was increased for 520 folds. However, INA facilitates the killing (Figure 2E).

To investigate such synergistic effect works *in vivo*, bacterial loads were monitored in different tissue of mice. After bacterial challenge, the treatment of saline, INA or TIG alone could not or only slightly reduced bacterial loads in liver, kidney and spleen, but when both of INA and TIG were present, the bacterial loads were significantly reduced in the tissues (Figure 2F). These data together suggest that INA potentiate TIG killing both *in vitro* and *in vivo*.

### INA enhances TIG bacteriostatic activity

**Figure 3.**
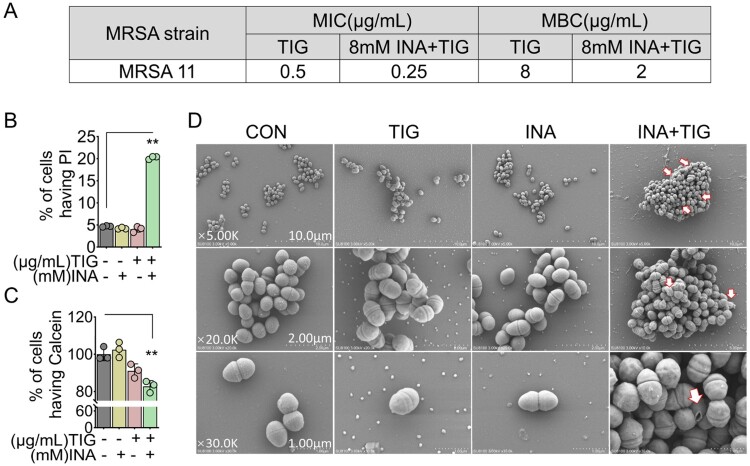
INA enhances TIG bacteriostatic activity. (A) MIC and MBC of MRSA11 in the presence or absence of INA. (B) Percent of cells having PI of MRSA11 in the absence or presence of INA plus TIG. (C) Percent of cells having calcein of MRSA11 in the absence or presence of INA plus TIG. (D) Scanning electron micrographs of MRSA11 being treated with INA, TIG, or both (Scalar bar = 1, 2, 10 μm, respectively). Results are displayed as mean ± standard errors of the means (SEM) (*N* ≥ 3 technical replicates per sample), and statistically significant differences are identified by Student’s *t* test. *, *p* < 0.05, **, *p* < 0.01. Each experiment was repeated independently at least three times.

TIG is known as bacteriostatic antibiotic by stalling cellular activity but not directly causing cell death [40]. But higher concentration of bacteriostatic antibiotic can cause cell death [41]. To explore whether INA affects TIG bacteriostatic activity, we measured the minimal bactericidal concentration (MBC) of INA on TIG using the MRSA11. The MIC of MRSA11 to TIG was 0.5 μg/mL, and the MBC was 8 μg/mL. The difference between MIC and MBC was 16 folds, confirming that TIG was bacteriostatic antibiotics. Whereas the presence of INA at 8 mM decreased MIC of MRSA11 from 0.5 to 0.25 μg/mL, and the MBC of TIG dropped from 8 to 2 μg/mL, where the difference was four folds (Figure 3A). These data suggest that TIG is a bacteriostatic antibiotic even in the presence of INA.

The antimicrobial activity of INA and TIG was further analysed at cellular level. Membrane permeability was investigated with propidium iodide (PI), which can only cross the dead cell [42]. Treatment with TIG or INA alone had few PI positive cells, while the number of PI positive cells was increased when supplemented with both of TIG and INA (Figure 3B). Calcein leakage assay can be used to determine the membrane integrity [43,44]. Similarly, TIG had no effect of calcein leakage. TIG alone reduced calcein positive cell, while INA plus TIG further decreased the number of calcein positive cell (Figure 3C). In addition, scanning electron microscope (SEM) analysis showed that the INA or TIG had no obvious effect on membrane structure, whereas the INA plus TIG cause membrane rupture, and even some bacteria membrane was broken (Figure 3D). These data together suggest that INA enhances the killing of TIG to MRSA and possibly through membrane integrity.

### INA enhances intracellular accumulation of TIG

**Figure 4.**
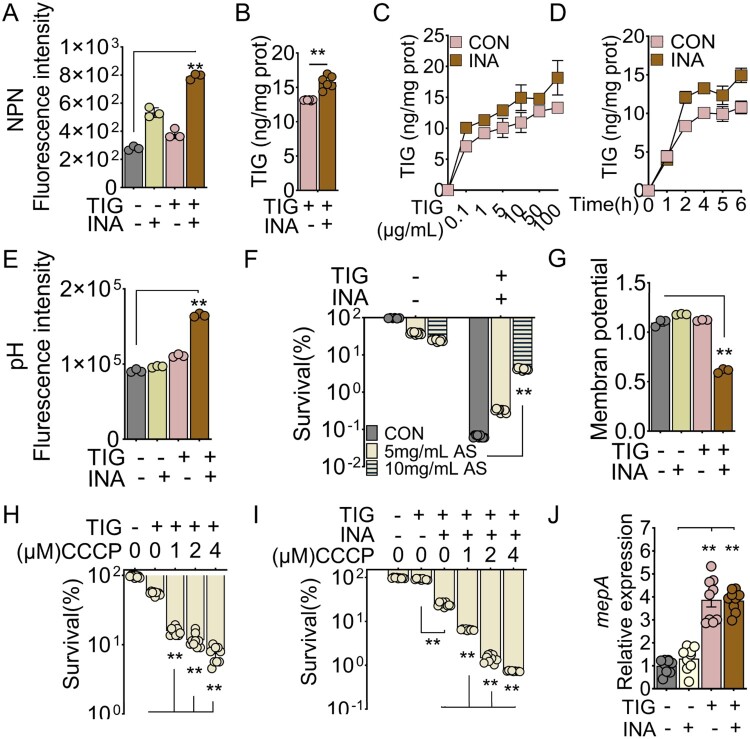
INA enhances intracellular accumulation of TIG. (A) NPN fluorescence intensity of MRSA11 in the presence or absence of 8 mM INA plus 0.04 μg/mL TIG. (B) Intracellular TIG of MRSA11 being treated with indicated concentrations of 8 mM INA plus 0.04 μg/mL TIG. (C) Intracellular TIG of MRSA11 being treated with INA plus the increasing concentration of TIG. (D) Intracellular TIG of MRSA11 being treated with INA plus TIG at the indicated incubation periods. (E) pH fluorescence intensity of MRSA11 in the presence or absence of 8 mM INA plus 0.04 μg/mL TIG. (F) Percent survival of MRSA11 in the presence of the increasing concentration of AS with INA plus TIG. (G) Membrane potential of MRSA11 in the presence or absence of 8 mM INA plus 0.04 μg/mL TIG. (H, I) Percent survival of MRSA11 in the presence of the increasing concentration of CCCP with INA plus TIG. (J) qRT-PCR for expression of *mepA* of MRSA11 in the absence and presence of INA plus TIG. Results are displayed as mean ± standard errors of the means (SEM) (*N* ≥ 3 technical replicates per sample), and statistically significant differences are identified by student’s *t* test. *, *p* < 0.05, **, *p* < 0.01. Each experiment was repeated independently at least three times.

Intracellular concentration of antibiotics is crucial for antibiotic function. To investigate whether INA could enhance antibiotic influx into bacteria, we first determined the change of membrane permeability. MRSA uptake of N-phenyl-1-napthylamine (NPN), a fluorescence probe, was performed in the presence of INA, TIG or both. Treating MRSA11 with INA or TIG alone slightly increased NPN uptake, whereas when both of INA and TIG were present, the uptake was increased for 2.83 folds (Figure 4A). Thus we determined the intracellular TIG concentration. Similar to the NPN uptake assay, intracellular concentration of TIG was highest in the bacteria being treated with INA and TIG as compared to TIG alone (Figure 4B). Furthermore, intracellular concentration of TIG was increased along with the increasing extracellular concentrations of TIG and the presence of INA increased intracellular TIG (Figure 4C). Similarly, intracellular TIG was also increased in a time-dependent manner and was enhanced by the presence of INA (Figure 4D). These results demonstrate that INA enhanced TIG accumulation in the bacteria.

Intracellular TIG concentration was affected by dynamic antibiotic influx and efflux. Proton motive force (PMF) plays critical roles in regulating TIG dynamics in bacteria, which is composed of two components, a transmembrane electrical potential and a transmembrane pH gradient [45]. pH gradient regulates TIG influx whereas membrane potential enhances TIG efflux [46,47]. To investigate whether INA affects these two components. MRSA was treated with TIG or INA or both. For pH gradient, the fluorescence intensity was slightly increased by TIG or INA but was significantly increased by both, suggesting enhanced influx of TIG in the presence of INA (Figure 4E). Ammonia sulphate (AS), which disrupts the pH gradient, decreased the synergistic effect of TIG and INA in a dose-dependent manner (Figure 4F).

In the contrast, the membrane potential showed a completely different pattern. INA alone decreased the membrane potential while TIG increased the membrane potential. More importantly, INA counteracted the increased potential induced by TIG, lower than the non-treated control (Figure 4G). Carbonyl-cyanide 3-chlorophenylhydrzone (CCCP) is a known chemical to abrogate membrane potential [48,49]. The treatment of MRSA with CCCP enhanced TIG killing in a dose-dependent manner, confirming INA-decreased PMF were critical for TIG killing (Figure 4H). And CCCP had additive effect to INA on TIG killing activity than CCCP alone (Figure 4I). In addition, MepA, encoded by *mepA*, is an efflux pump in MRSA, whose overexpression or mutation causes TIG resistance [50]. When quantified the gene expression of this gene, TIG treatment significantly increased *mepA* expression but not by INA alone. Interestingly, INA plus TIG had no significant effect on the expression as compared to TIG (Figure 4J). Thus TIG increases *mep* expression but not affected by the presence of INA. For other efflux pumps in *S. aureus* that had not yet reported to confer TIG resistance, only the expression of *norA* and *norB* (efflux pumps for fluoroquinolones in MRSA), MDR and *tet38* were increased by TIG but not further boosted by INA (see Suppl. Figure 4). These data together suggest that INA promotes intracellular TIG accumulation by promoting drug influx but limiting efflux dependent on PMF.

### INA plus TIG destabilize cellular membrane to promote TIG accumulation

**Figure 5.**
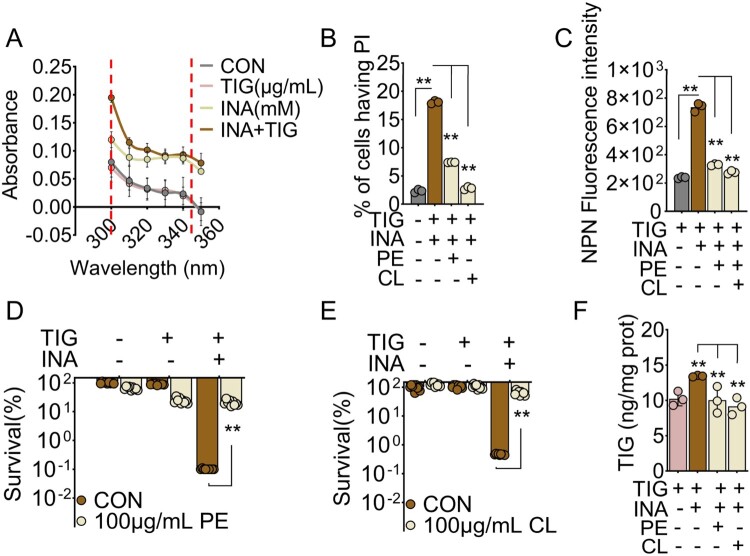
INA plus TIG destabilize cellular membrane to promote TIG accumulation. (A) INA-NAD adduct of MRSA11 in the presence or absence of 8 mM INA plus 0.04 μg/mL TIG. (B) Percent of cells having PI of MRSA11 in the absence or presence of PE or CL with 8 mM INA plus 0.04 μg/mL TIG. (C) NPN fluorescence intensity of MRSA11 in the absence or presence of PE or CL with 8 mM INA plus 0.04 μg/mL TIG. (D, E) Percent survival of MRSA11 in the absence or presence of PE or CL with 8 mM INA plus 0.04 μg/mL TIG. (F) Intracellular TIG of MRSA11 in the absence or presence of PE or CL with INA plus TIG. Results are displayed as mean ± standard errors of the means (SEM) (*N* ≥ 3 technical replicates per sample), and statistically significant differences are identified by student’s *t* test. *, *p* < 0.05, **, *p* < 0.01. Each experiment was repeated independently at least three times.

**Figure 6.**
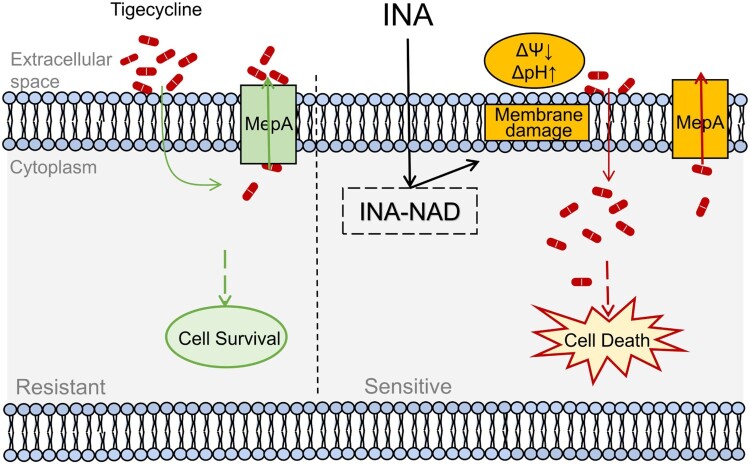
Proposed model. INA enhances the TIG influx to enhance intracellular TIG accumulation to combat MRSA infection.

To explore how INA plus TIG promote TIG accumulation, we first determine the formation of INA-NAD adduct, a functional form of INA. As compared to TIG alone, INA or INA plus TIG increased the formation of INA-NAD, where INA plus TIG induced higher levels of adduct (Figure 5A). INA-NAD is highly reactive and could damage different cellular structure [18,19]. Our above results suggest that INA plus TIG alter membrane permeability (Figures 3C and D). The addition of phosphatidylethanolamine (PE) and cardiolipin (CL), the key components of phospholipids in bacterial membrane abrogating the effect of membrane-targeting drugs [47,51], could decrease membrane permeability that was induced by INA plus TIG, where the percent of PI positive cells was dropped from 18.32% to 7.44% by PE and to 3.08% by CL (Figure 5B). Similarly, PE and CL also reduced NPN by INA and TIG (Figure 5C). Being consistently, PE and CL increased bacterial survival for 228.3 and 114.17 folds in the presence of both of INA and TIG (Figures 5D and E). However, PE and CL had limited protective effects to TIG. Furthermore, when bacteria were treated with INA plus TIG in the presence of PE and CL, PE and CL decreased intracellular TIG concentration to a similar level of TIG alone (Figure 5F). Moreover, the PE and CL abrogated INA-induced change on membrane potential and pH, suggesting bacteria restore resistance to TIG. Importantly, INA didn’t interact with PE and CL as assayed by ITC (see Suppl. Figure 5). Taken together, these data suggest that INA form INA-NAD adduct that destabilize membrane to allow the influx but limit efflux of TIG (Figure 6).

## Discussion

The rising of antibiotic resistance has been subjected to big concerns both in academic and society. The urgent need for the development of strategies in dealing with the situation leads to two major routes, the discovery of new antibiotics and the search for antibiotic adjuvants that potentiate current available antibiotics [52,53]. Antibiotic combinations are of such strategy that is believed to rejuvenate the efficacy of antibiotics to which bacteria have developed resistance, avoid toxicity but getting greater therapeutic effect, and shorten the duration of therapy but with fast and low-cost development [54].

In this study, we exploit the antibiotic combinations of INA and TIG in treating MRSA. INA is a prodrug that is converted to its active form through KatG, catalase-peroxidase enzyme, in MTB [55]. The active form of INA can affect multiple cellular process that contributes to bactericidal activity of INA [19]. Although *katG* gene is widely present across different bacterial species, INA has poor activity against other types of bacterial pathogens. Meanwhile, it is possible that using INA as a molecule to remodel bacterial metabolism to enhance the activity of other types of antibiotics. Thus we explore such possibility by testing the combination of INA with various types of antibiotics, and we found it synergize best with TIG. TIG belongs to the tetracycline class that can be used to treat polymicrobial multidrug-resistant infection by both of gram-positive and gram-negative bacteria [16]. TIG has been proved to be effective against not only MRSA but also vancomycin-resistant *enterococci*, and extended-spectrum β-lactamase (ESBL)-producing *Enterobacteriaceae* [56]. A recent meta-analysis of TIG resistance in MRSA suggests that TIG should be used in caution to prevent the occurrence of more resistance [13], e.g. 3% resistance rate of MRSA in India and 53.5% isolates of *S. aureus* are non-susceptible to TIG [57]. Thus the identification of INH as an antibiotic adjuvant is expected to increase the lifespan of TIG in clinic.

The doses we used in this study are clinic relevant. Our bacterial challenge assay employs 10 mg/kg TIG or/and 100 mg/kg INA. The dose used in mouse can be translated into human dose based on surface area with a coefficient of 0.081 [58]. Thus the corresponding dose used in mice is equivalent of 0.81 mg/kg for TIG or/and 8.1 mg/kg for INA. The recommended dose regimen for TIG is 100 mg at initial dose and followed by 50 mg for every 12 h [56]. To treat MTB in children, patients received 10.2 mg/kg for intermittent regimen or 8.4 mg/kg for daily regimen [59]. The standard dose of INA used in adult is 5–15 mg/kg depending on the duration of treatment [60]. Moreover, INA may also act through immune system to facilitate the elimination of MTB infection. INA induces progenitor stem cell differentiate into pro-inflammatory monocytes, thus increasing monocyte population, and strengthen the immune competency of granuloma [61]. Additionally, INA-induced autophagy in infected immune cell, and inhibiting IL-1R1 and TNF signalling are required for the antimycobacterial function of INA [62,63]. However, whether INA in combing with TIG also act through immune system, and whether INA has a broad immune response-promoting effects should be explored.

Antibiotic influx is the key to reverse antibiotic resistance. We demonstrate that INA drive the TIG influx into the bacteria that are the key to enhance the antibacterial activity of TIG. Removing the membrane barrier to allow the antibiotic enter into the cell is considered as a major challenge in combating antibiotic resistance because of antibiotic-resistant bacteria has low cell permeability [27,64]. The rational design of small molecule, e.g. colistin, to disrupt is believed to enhance the killing of bacteria [65]. Our study suggests that INA itself has low toxicity to MRSA and can be a potential TIG adjuvant to MRSA infection. Furthermore, the recent advance on metabolic regulation of antibiotic resistance also enlightens the use of metabolites to promote antibiotic influx. Alanine, glucose, fructose, glutamate and pyruvate enhance intracellular accumulation of aminoglycosides through the pyruvate cycle [64,66]. Glutamine and fructose promote β-lactams influx into the cell [21,33]. Although these studies were performed mainly in gram-negative bacteria, this study that developing approach to enhance intracellular antibiotic accumulation is also key to combat MRSA infection.

Although we demonstrate that INA and TIG are potential on eliminating MRSA infection in a mouse infection model, we have to note that this infection model only represents bacteraemia. MRSA also commonly causes other infection like skin and soft tissue, and joint infections. Despite that the synergistic effect should be investigated in different infection model to show its use in different clinic settings in the future, we do believe the boosting effect of INA to TIG. The reason is because of the mechanism that INA mainly promotes MRSA uptake of TIG. A previous study showed that glutamine increased bacteria uptake of ampicillin that help mouse defence against different bacterial pathogens [33]. Thus INA and TIG could also be possibly effective against MRSA infection in other clinic-relevant situation.

In conclusion, our study identifies INA is not only an anti-MTB antibiotic but also can be explored as an antibiotic adjuvant to antibiotics to kill non-MTB bacterial pathogens. And the main function of INA in revering antibiotic resistance of MRSA is to enhance the TIG influx. Thus our study suggests a new approach to control MRSA infection through antibiotic combinations.

## Supplementary Material

Supplementary Materials.docx
