# Supplementary material for: Isoniazid potentiates tigecycline to kill methicillin-resistant Staphylococcus aureus
Source: Emerg Microbes Infect. 2024 Nov 25;14(1):2434587. doi: 10.1080/22221751.2024.2434587 (PMC12093414; doi:10.1080/22221751.2024.2434587)
Supplement: Supplementary Materials.docx [file TEMI_A_2434587_SM5690.docx]

**Supplementary material**


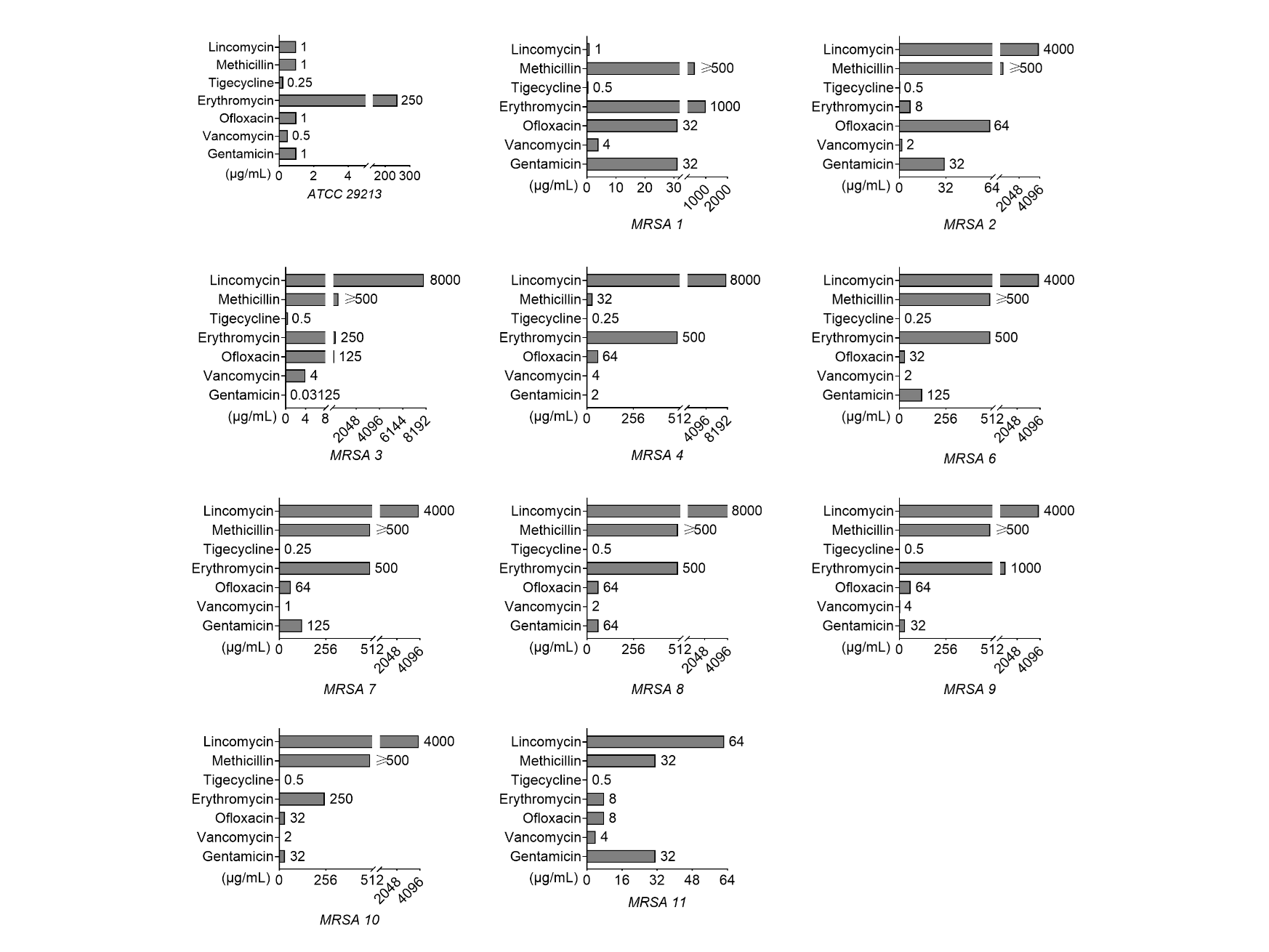


**Figure 1**. Antimicrobial profiles of the standard strain, ATCC29213, and 10 MRSA strains to lincomycin, methicillin, tigecycline, erythromycin, ofloxacin, vancomycin and gentamycin.


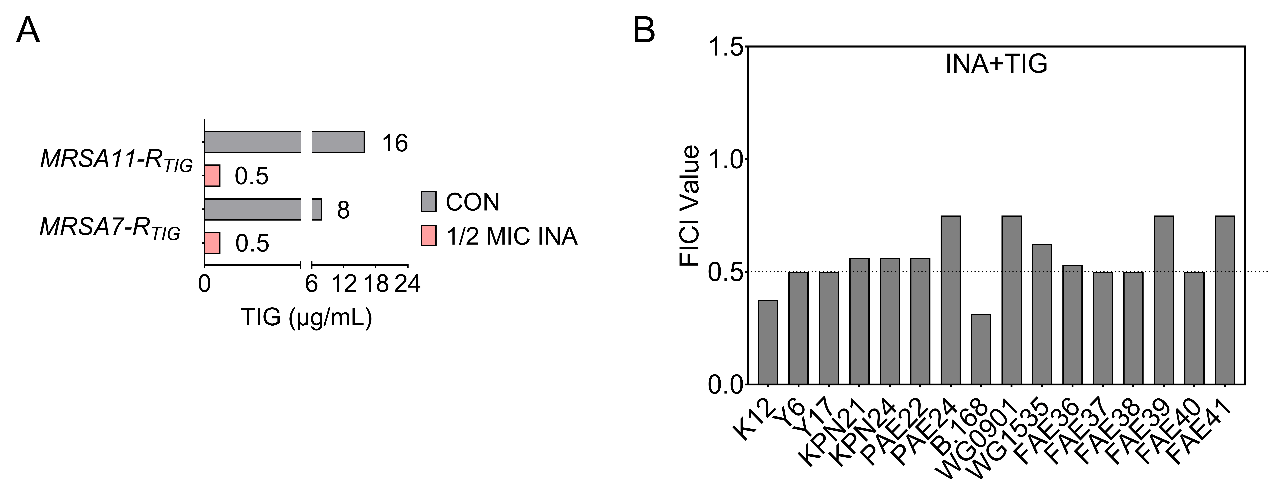


**Figure 2.** Synergistic effect of INA and TIG on lab-evolved TIG-resistant MRSA and other bacterial strains. (A) MIC of MRSA7-R_TIG_ and MRSA11-R_TIG_ in the presence of absence of 1/2 MIC INA. MRSA7-R_TIG_ and MRSA11--R_TIG_ were obtained by evolving MRSA7 and MRSA11 in the presence of TIG_._ (B) FICI value of other pathogens including *E. coli* (K12, Y6, Y17), *K. pneumonia* (KPN21, KPN24), and *P. aeruginosa* (PAE22, PAE24)*, B. subtilis* (B.168), *S. agalactiae* (WG0901, WG1535) *E. faecium* (FAE36, FAE37, FAE38, FAE39, FAE40, FAE41).


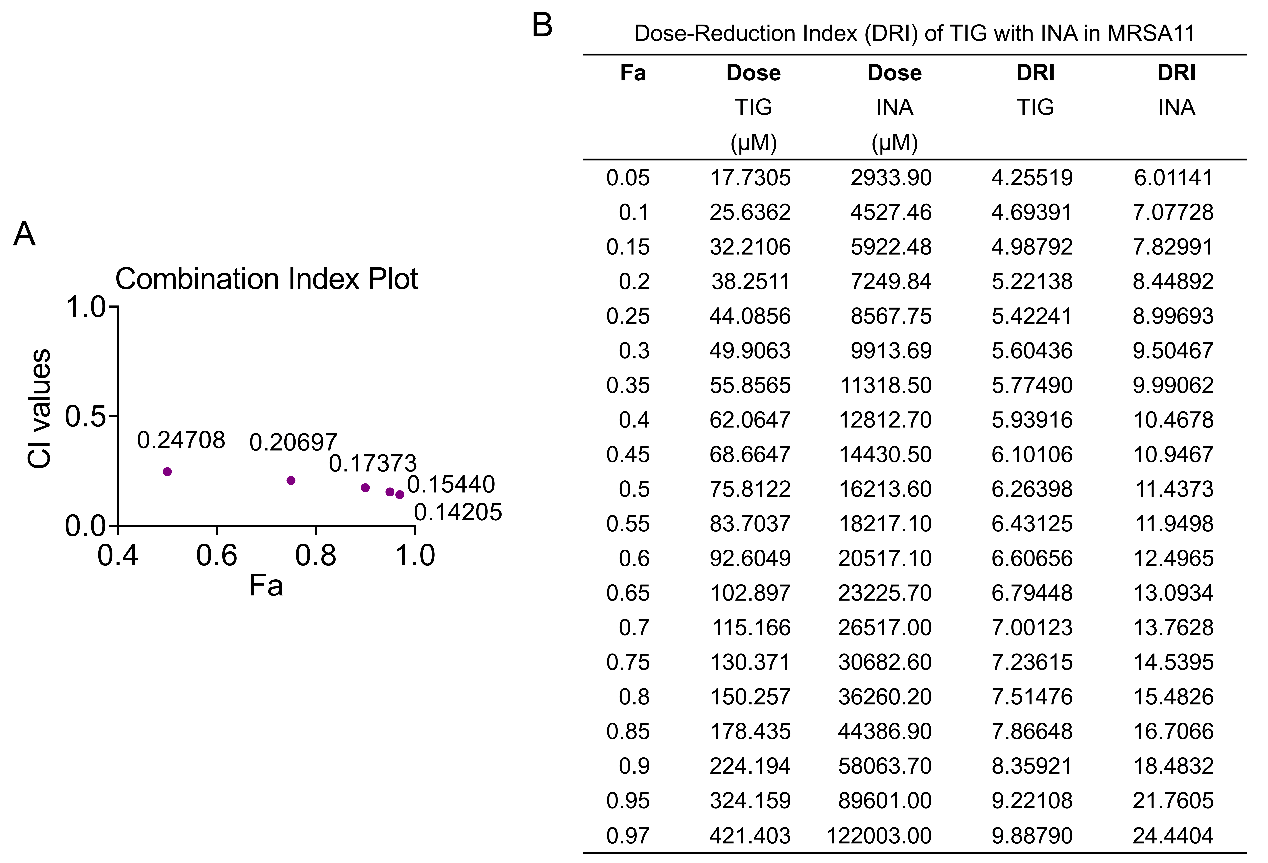


**Figure 3.** Combination index plot (A) and dose-reduction index (B) for drug combinations of INA and TIG. F_a_ is the fraction affected by the dose.


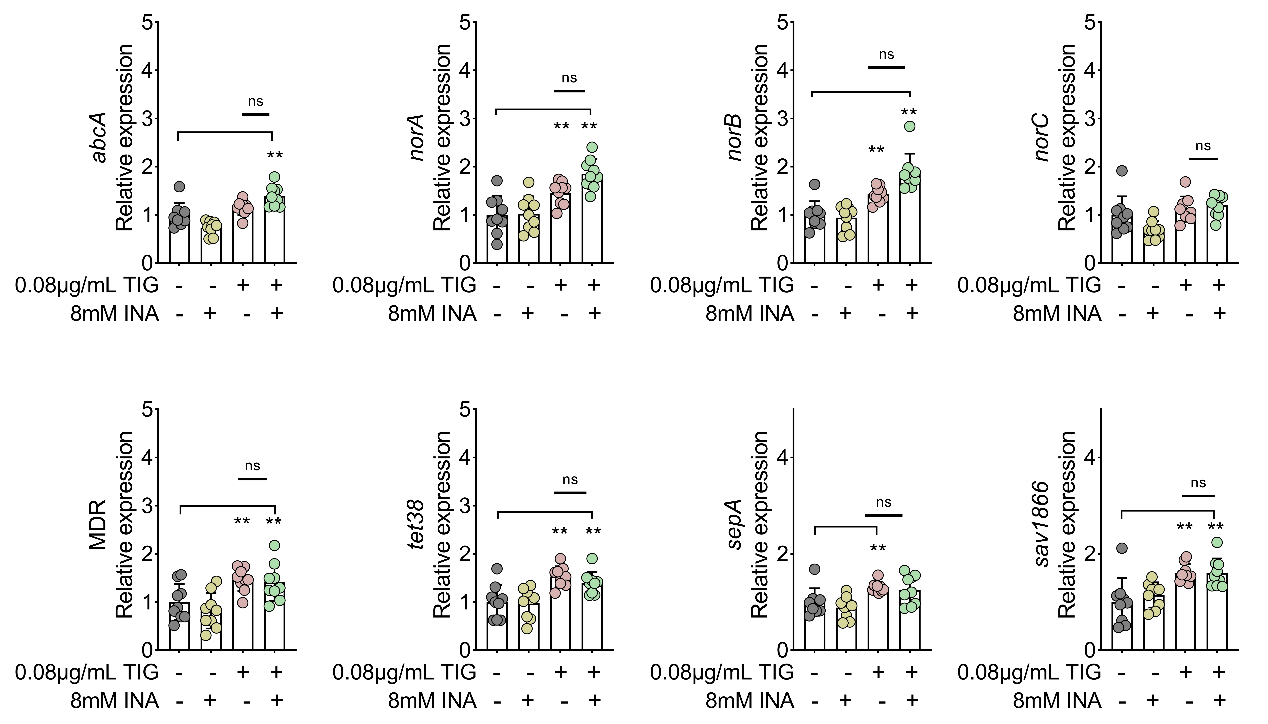


**Figure 4.** qRT-PCR for expression of efflux pumps in the absence and presence of INA plus TIG. Results are displayed as mean ± standard errors (SEM) (N≥3 technical replicates per sample), and statistically significant differences are identified by Student’s t test. *, p< 0.05, **, p< 0.01. Each experiment was repeated independently at least three times.


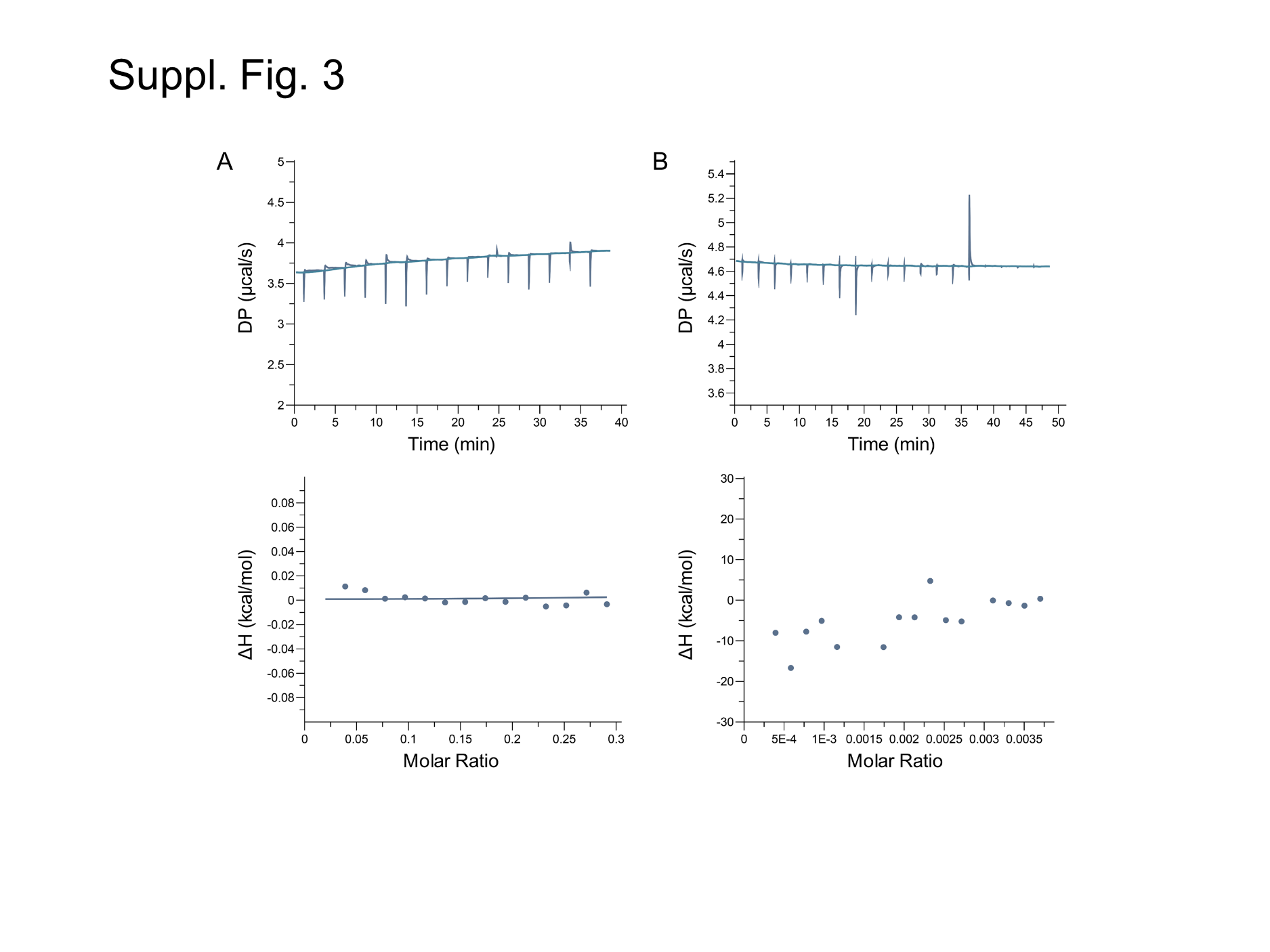


**Figure 5**. ITC assay of the binding of INA to PE (A) or CL (B).

**Supplementary Table 1. Primers used in this study**

| Gene（Primer） | Nucleotide Sequence (5′-3′) |
| --- | --- |
| *mepA-F* | ACGAACAGGTCCAACTGTCA |
| *mepA-R*  *abcA-F*  *abcA-R*  *norA-F*  *norA-R*  *norB-F*  *norB-R*  *norC-F*  *norC-R*  *tet38-F*  *tet38-R*  *SAV1866-F*  *SAV1866-R*  *sepA-F*  *sepA-R*  *MDR-F*  *MDR-R* | GCGATACGAGTGTTTGCTCC  AACCCGACAGGTCGTTCTTT  TGATAGCAGGAGTTGGATCGC  TATCGCCGTTTGGTGGTACG  AACCTGTCACACCAGGCATT  TTAGGTGGAGCATTTGGCGT  GCCATTCCTGCGTTTAACCAT  GCGGGGGCAACAAGTTAATC  TGTTCCCATGTCTGCACCAA  TCGTTGGTGCACTAAGTGGT  AGCAAAGCTACCTGCAAAGA  TTCGGGCCTTTACGTCGTTT  AGGTTGAGCACCAACACCAT  TGCAGTCGAGCATTTAATGGA  ACGTTGTTGCAACTGTGTAAGA  AGCCATTGTTGAGATGGCGA  GGCCATAATTGTAGCACCGC |
